# Detecting Periodic Genes from Irregularly Sampled Gene Expressions: A Comparison Study

**DOI:** 10.1155/2008/769293

**Published:** 2008-06-04

**Authors:** Wentao Zhao, Kwadwo Agyepong, Erchin Serpedin, Edward R Dougherty

**Affiliations:** 1 Department of Electrical and Computer Engineering, Texas A&M University, College Station, TX 77843, USA; 2 Genomics Research Institute, 400 North Fifth Street, Suite 1600, Phoenix, AZ 85004, USA

## Abstract

Time series microarray measurements of gene expressions have been exploited to discover genes involved in cell cycles. Due to experimental constraints, most microarray observations are obtained through irregular sampling. In this paper three popular spectral analysis schemes, namely, Lomb-Scargle, Capon and missing-data amplitude and phase estimation (MAPES), are compared in terms of their ability and efficiency to recover periodically expressed genes. Based on *in silico* experiments for microarray measurements of *Saccharomyces cerevisiae*, Lomb-Scargle is found to be the most efficacious scheme. 149 genes are then identified to be periodically expressed in the *Drosophila melanogaster* data set.

## 1. Introduction

The functioning of eukaryotic cells is controlled by accurate timing of biological cycles, such as cell cycles and circadian rhythms. These are composed of an echelon of molecular events and checkpoints. At the transcription level, these events can be quantitatively observed by measuring the concentration of messenger RNA (mRNA), which is transcribed from DNA and serves as the template for synthesizing protein. To achieve this goal, in the microarray experiments, high-throughput gene chips are exploited to measure genome-wide gene expressions sequentially at discrete time points. These time series data have three characteristics. Firstly, most data sets are of small sample size, usually not more than 50 data points. Large sample sizes are not financially affordable due to high cost of gene chips. Also the cell cultures lose their synchronization and render meaningless data after a period of time. Secondly, the data are usually evenly sampled and have many time points missing. Thirdly, most data sets are customarily corrupted by experimental noise and the produced uncertainty should be addressed in a stochastic framework.

Extensive genome-wide time course microarray experiments have been conducted on organisms such as *Saccharomyces cerevisiae* (budding yeast) [1], human Hela [2], and *Drosophila melanogaster* (fruit fly) [3]. Budding yeast in [1] has served as the predominant data source for various statistical methods in search of periodically expressed genes, mainly due to its pioneering publication and relatively larger sample size compared with its peers. By assuming the signal in the cell cycle to be a simple sinusoid, Spellman et al. [1] and Whitfield et al. [2] performed a Fourier transformation on the data sampled with different synchronization methods, while Giurcaneanu [4] explored the stochastic complexity of the detection mechanism of periodically expressed genes by means of generalized Gaussian distributions. Ahdesmäki et al. [5] implemented a robust periodicity testing procedure also based on the non-Gaussian noise assumption. Alternatively, Luan and Li [6] employed guide genes and constructed cubic B-spline-based periodic functions for modeling, while Lu et al. [7] employed up to three harmonics to fit the data and proposed a periodic normal mixture model. Power spectral density estimation schemes have also been employed. Wichert et al. [8] applied the traditional periodogram on various data sets. Bowles et al. [9] compared Capon and robust Capon methods in terms of their ability to identify a predetermined frequency using evenly sampled data sets, under the assumption of a known period. Lichtenberg et al. [10] compared [1,6,7] while proposing a new score by combining the periodicity and regulation magnitude. The majority of these works dealt with evenly sampled data. When missing data points were present, either the vacancies were filled by interpolation in time domain, or the genes were discarded if there were more than 30% data samples missing.

Biological experiments generally output unequally spaced measurements. The major reasons are experimental constraints and event-driven observation. The rate of measurement is directly proportional to the occurrence of events. Therefore, an analysis based on unevenly sampled data is practically desired and technically more challenging. While providing modern spectral estimation methods for stationary processes with complete and evenly sampled data [11], the signal processing literature has witnessed an increased interest in analyzing unevenly sampled data sets, especially in astronomy, in the last decades. The harmonics exploited in discrete Fourier transform (DFT) are no longer orthogonal for uneven sampling. However, Lomb [12] and Scargle [13] demonstrated that a phase shift suffices to make the sine and cosine terms orthogonal. The Lomb-Scargle scheme has been exploited in analyzing the budding yeast data set by Glynn et al. [14]. Schwarzenberg-Czerny [15] employed one-way analysis of variance (AoV) and formulated an AoV periodogram as a method to detect sharp periodicities. However, it relies on an infeasible biological assumption, that is, the observation duration covers many cycles. Along with this line of research, Ahdesmäki et al. [16] proposed to use robust regression techniques, while Stoica and Sandgren [17] updated the traditional Capon method to cope with the irregularly sampled data. Wang et al. [18] reported a novel technique, referred to as the missing-data amplitude and phase estimation (MAPES) approach, which estimates the missing data and spectra iteratively through the expectation maximization (EM) algorithm. In general, Capon and MAPES methods possess a better spectral resolution than Lomb-Scargle periodogram. In this paper, we propose to analyze the performance of three of the most representative spectral estimation methods: Lomb-Scargle periodogram, Capon method, and the MAPES technique in the presence of missing samples and irregularly spaced samples. The following questions are to be answered in this study: do technically more sophisticated schemes, such as MAPES, achieve a better performance on real biological data sets than on simpler schemes? Is the efficiency sacrificed in using these advanced methods justifiable?

The remainder of this paper is structured as follows. In Section 2, we introduce the three spectral analysis methods, that is, Lomb-Scargle, Capon and MAPES. Hypothesis tests for periodicity detection and the corresponding -values are also formulated. The multiple testing correction is discussed. Section 3 presents simulation results. The performances of the three schemes are compared based on published cell-cycle and noncell-cycle genes of the Saccharomyces cerevisiae (budding yeast). Then the spectral analysis for the data set of Drosophila melanogaster (fruit fly) is performed, and a list of 149 genes are presented as cycle-related genes. The synchronization effects are also considered. Concluding remarks and future works constitute the last section, and full results are provided online in the supplementary materials [19].

## 2. Methods

In this section, the Lomb-Scargle periodogram, Capon method, and MAPES approach are introduced and compared in terms of their features and implementation complexity. The detailed derivations are omitted. As a general notational convention, matrices and vectors are represented in bold characters, while scalars are denoted in regular fonts.

## 2.1. Lomb-Scargle Periodogram

The deployment of Fourier transform and traditional periodogram relies on evenly sampled data, which are projected on orthogonal sine and cosine harmonics. The uneven sampling ruins this orthogonality. Hence, the Parseval's theorem fails, and there exists a power discrepancy between the time and frequency domains. When analyzing astronomical data, which in general are collected at uncontrollable observation times, Lomb [12] found that a phase shift of the sine and cosine functions would restore the orthogonality among harmonics. Scargle [13] complemented the Lomb's periodogram by exploiting its distribution. Since then, the established Lomb-Scargle periodogram has been exploited in numerous fields and applications, including bioinformatics and genomics (see, e.g., Glynn et al. [14]).

Given  time-series observations , , where  stands for the time tag and  denotes the sampled expression of a specific gene, the normalized Lomb-Scargle periodogram for that gene expression at angular frequency  is defined as(1)

where  and  stand for the mean and variance of the sampled data, respectively, and  is defined as(2)

For evenly sampled data, the sampling interval  can be expressed as(3)

The highest frequency, namely, the Nyquist frequency, is . Beyond this limit, the computed spectra repeat. For unevenly sampled data, a straightforward way to introduce the Nyquist frequency is by keeping the definition as in the evenly sampled case, that is, using the averaged sampling interval defined in the second equality of (3), as is employed in Glynn's work [14]. Actually, Eyer and Bartholdi in [20] proved that the highest frequency is much larger than . Let  be the greatest common divisor (gcd) for all intervals  (), then the highest frequency that should be searched is given by(4)

The number of probing frequencies is denoted by(5)

and the frequency grid can be defined in terms of the following equation:(6)

Notice further that the spectra on the front and rear halves of the frequency grid are symmetric since the microarray experiments output real values.

Lomb-Scargle periodogram represents an efficient solution in estimating the spectra of unevenly sampled data sets. Our simulation results also verify its superior performance for biological data with small sample size and various unevenly sampled patterns.

## 2.2. Capon Method

Capon method represents a modern power spectral estimation technique that yields better spectral resolution compared with traditional periodogram [11]. The original Capon method tries to design a filter-bank by taking properties of its data into account. Assuming  observations are equally spaced with a sampling interval , at a frequency , the Capon filter is designed so that the power of the filter's output is minimized while the frequency  is passed without distortion. Solving this optimization problem provides the spectrum estimate at frequency  as(7)

where the  stands for the data covariance matrix with a dimension , which is also the bandwidth of the Capon filter. The ancillary vector is defined as follows:(8)

Note that we have not included in this spectrum estimate a scaling factor. However, the absence of this scaling factor does not affect periodicity analysis for the genes. Therefore, we neglect this scaling factor. The bandwidth parameter  cannot exceed  to guarantee an existing . The larger the , the better the resolution of the obtained spectra.

Recently, the Capon method has been updated to cope with the presence of irregular samples [17]. The same frequency grid denoted in (6) is employed. The  has to be changed to , the greatest common divisor between any two sampling times. In order to take advantage of the best resolution,  is set to be equal to , where  is defined in (5). In our simulation, an estimate of the autocorrelation matrix  can be obtained from the Lomb-Scargle periodogram. It can be represented by(9)

The Capon method is slightly more computationally complex than Lomb-Scargle periodogram, and it usually achieves a better performance in terms of resolution provided that there are sufficient samples. However, for highly corrupted biological data with small sample size, this is not true.

## 2.3. MAPES Method

Regular sampling can be treated as a case of missing data as long as the sampling time tags share a greatest common divisor. This constraint is satisfied in most biological experiments and published data sets. The missing-data amplitude and phase estimation (MAPES) method, proposed in [18], is a nonparametric spectral estimation approach. It is robust to error modeling and it deals with arbitrary data-missing patterns as opposed to gapped or periodically gapped data, and achieves a better spectral resolution in the sense of resolving closely spaced spectral lines. However, the exploitation of the expectation maximization (EM) algorithm sacrifices its computational efficiency.

The data, , , are assumed to be sampled uniformly, however, only  data points are available and there are  missing data points. Noticeably,  still conforms to the definition in (5). The gene expression signal with frequency  can be modeled as(10)

where  represents the complex amplitude of the sinusoidal component and  denotes the residual term. The probing frequencies still follow (6). Employing the EM algorithm, MAPES tries to iteratively assess the missing data, and meanwhile to update the estimation of spectra and error.

The data vector  can be partitioned into  overlapping subvectors, each with dimension , and . These subvectors constitute the enhanced data vector , which assumes the following expression:(11)

where  and  represent the available and missing data, respectively, and  and  denote their selection matrices, respectively. Alternatively, given , , and , the data vectors ,  can be computed in the least-square (LS) sense as follows:(12)

The residual vector and its covariance matrix are next defined(13)

where  denotes the expectation operator, and in practice is replaced by a sample mean estimator. The following two notations are also required by the definition of MAPES power spectral estimator:(14)

In the th EM iteration, the probability density function (PDF) of the missing data vector  conditioned on the available data  and other context parameters is complex Gaussian with mean and variance denoted by  as follows:(15)

Then the estimates for spectral magnitude  and residual matrix  are updated in terms of equations(16)

where the auxiliary matrices are defined as follows:(17)(18)(19)

In (19),  are  subblock matrices located on the main diagonal of matrix .

Finally, the MAPES power spectral density estimator can be expressed as(20)

Actually, in our *in silico* experiments, assuming , MAPES yields an estimate of power spectral about two orders of magnitude more computational time (roughly about one hundred times slower) than Lomb-Scargle and Capon methods. Also, the simulation results do not indicate any performance improvement for MAPES in terms of the ability to discover published cell cycle genes. A more detailed comparison between these schemes will be presented in the simulation section.

## 2.4. Periodicity Test

Based on the obtained power spectral density, each gene is to be classified as either a cyclic gene or noncyclic one. The null hypothesis is usually formed to assume that the measurements are generated by a Gaussian noise stochastic process. For a general periodogram or power spectral density estimator , Fisher's test can be exploited to examine the significance of the detected peak. The Fisher's test statistic is defined as(21)

where  since the spectra on the defined frequency grid are symmetric. The -value for detecting the largest peak is given by [21](22)

A rejection of the null hypothesis based on a -value threshold implies that the power spectral density contains a frequency with magnitude substantially greater than the average value. This indicates that the time series data contain a periodic signal and the corresponding gene is cyclic in expression. Notice also that a more accurate estimation method for the -values can be found in Fisher [22] or Brockwell and Davis [23]. The rank of genes ordered by their -values is of additional importance and it helps to hedge the risk of dichotomous decisions.

For the Lomb-Scargle periodogram,  is exponentially distributed under the null hypothesis [13], a result which is also exploited in [14]. However, this exponential distribution is not applicable for a general power spectral density. Therefore, Fisher's test is employed to perform the comparison among different spectral schemes. Our simulation results also show that for Lomb-Scargle periodogram, the gene ranks generated by Fisher's test do not differ much from that produced by the exponential distribution. Finally, we remark that other periodicity detection tests exist, as indicated by the robust Fisher test [24], the likelihood ratio test, and the  test [21].

## 2.5. Multiple Testing Correction

In order to prevent the false positives from overwhelming the true positives, the multiple testing correction, as proposed in [25,26], is performed to control the false discovery rate (FDR). For each of measured  genes, the periodicity is tested and a -value is generated. All -values are sorted in ascending order with the smallest th -value denoted by . Assume an estimate to the number of noncyclic genes among all  genes is , and the testing procedure preserves  genes which have lowest -values, then an estimate of FDR can be formulated as(23)

where the numerator is an estimate of the number of false positives. Since generally periodic genes only occupy a small portion of all genes, the  is set to  directly in our simulation. Such an action brings a slightly larger estimate. There exist other statistical methods to estimate , for example, [26].

The  is not a monotonic function of , the number of preserved genes. This property makes it tough to choose a -value threshold. To combat this, the -value is proposed in [25] as following:(24)

The -value is a monotonically increasing function with respect to . The FDR can be controlled via specifying the -value threshold as , through which the number of genes to preserve can then be derived as(25)

## 3. Simulation Results

Our *in silico* experiments are first performed on the *Saccharomyces cerevisiae* (budding yeast) data set. The Lomb-Scargle, Capon, and MAPES are compared. Then we proceed to analyze the *Drosophila melanogaster* (fruit fly) data set.

## 3.1. Simulation on Saccharomyces Cerevisiae

The performance of the three schemes is evaluated based on the *Saccharomyces cerevisiae* (budding yeast) data set reported by Spellman et al. [1]. In the biological experiments, the mRNA concentrations of more than 6 000 open reading frames (ORFs) were measured for the yeast strains synchronized by using four different methods, namely,  factor, cdc15, cdc28, and elutriation. The data set contained 73 sampling points, while there existed missing observations for some genes.

The literature has provided prior knowledge about the yeast cell cycle genes: Spellman et al. [1] enumerated 104 cell cycle genes that were verified in previous biological experiments, while Lichtenberg et al. [27] summarized 105 genes that were not involved in the cell cycle. By exploiting these two control sources, we can evaluate the true and false positives generated by the three spectral estimation methods.

The comparison procedure is as follows: based on the given data set, the three schemes perform to preserve a prespecified number of genes. These genes are marked as cell cycle genes and are compared with two control gene sets, from which the number of positives are counted. If a preserved gene also exists in the gene set which has been verified to be cell cycle regulated, this hit is counted as a true positive. On the other hand, if the preserved gene appears in the gene set which has been corroborated to be not involved in the cell cycle, this hit is counted as a false positive. Notice that since we expect the noncell cycle genes to be the majority of all measured genes, but the verified noncell cycle genes are only a small portion of all the genes, the false positives from verified noncell cycle genes only provide a reference but not a significant knowledge of the false positives. Because the three algorithms perform similarly for all four data sets, only simulation outcomes for cdc15 are presented here to exemplify the general results. The cdc15 data set contained 24 time points sampled from  minutes to  minutes. The greatest common divisor (gcd) for all time intervals is  minutes. Therefore  and . The bandwidth  of Capon method is 14 while the subvector length  of MAPES is equal to . All three schemes, that is, Lomb-Scargle, Capon, and MAPES, are applied on the data set.

**Figure 1:**
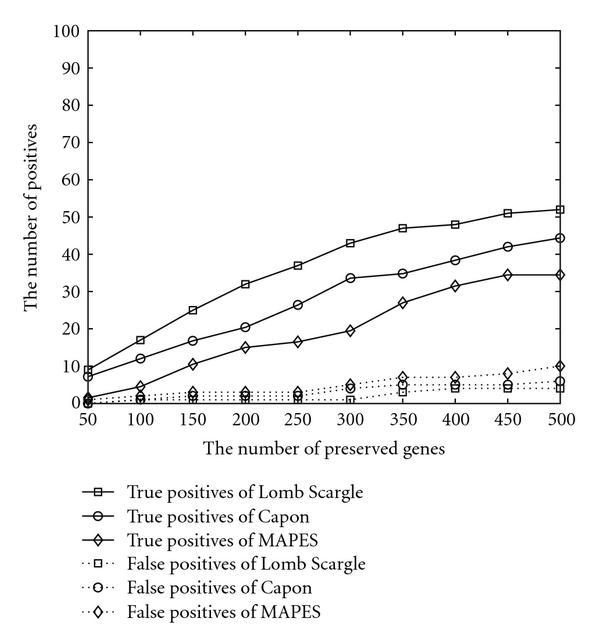
**Performance comparison based on cdc15 data set**.

The *in silico* results based on cdc15 data set are illustrated in Figure 1. When the number of preserved genes increases, all three schemes increase their ability to identify more cell cycle genes with more false discoveries as a tradeoff. Lomb-Scargle achieves the best performance in terms of identifying the highest number of true positives and producing lowest number of false positives, while MAPES was the worst with respect to these two metrics.

**Figure 2:**
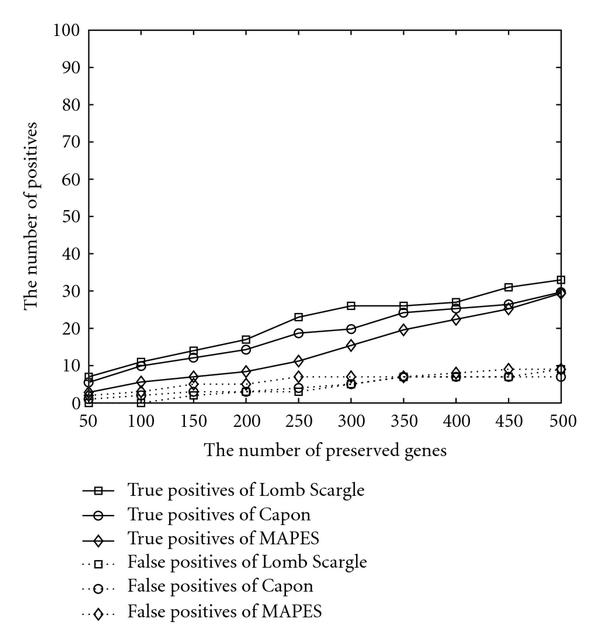
**Performance comparison when one third of measurements is randomly set to be missing**.

**Figure 3:**
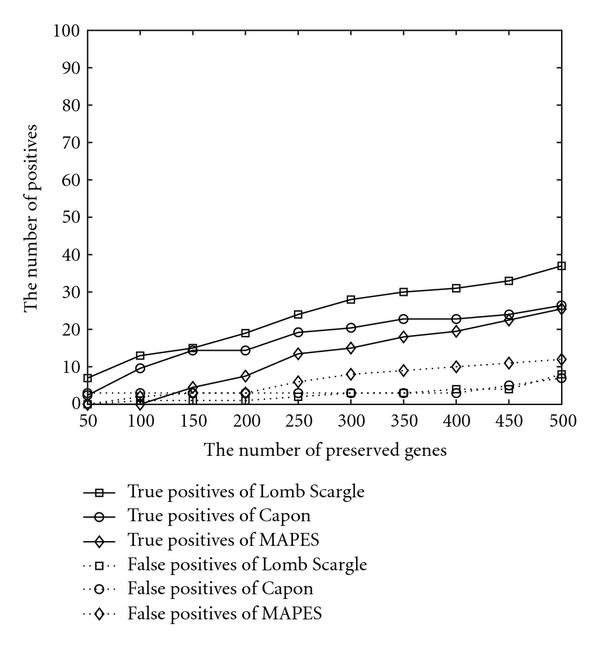
**Performance comparison when noise is intentionally added**.

To test the algorithm performance on the highly corrupted data, two *in silico* experiments are performed. Firstly, one third of all measurements is randomly set to be missing. The results are organized in Figure 2. Secondly, a gene's sampled data are added with Gaussian noise of mean 0 and variance equal to half of variance of the gene's measurements. The outcomes of the artificially noised data are presented in Figure 3. Compared with Figure 1, all of them identify less verified genes due to the artificially added noise or missed data. The false positives are controlled at a low level. The three algorithms behave in a similar pattern with respect to the increasing number of preserved genes.

Above all, Lomb-Scargle scheme always identifies the largest number of cell cycle genes that have been verified in previous biological experiments. Due to its simplicity, we recommend the use of this simplest method.

## 3.2. Simulation on Drosophila Melanogaster

The *Drosophila melanogaster* (fruit fly) is selected as our research target because it is a well-studied, relatively simple organism with a short generation time and only 4 pairs of chromosomes. In addition, 75% of human diseases have their counterparts in fruit fly, and 50% of fruit fly proteins have their mammalian analogs [28]. These make the fruit fly an excellent model for the research of human diseases. In the literature for the fruit fly, most of the research work was conducted through experimental biological methods, and the computational analysis tools have not been fully explored for the detection of periodically expressed genes. Our *in silico* experiments are performed on the fruit fly data set published by Arbeitman et al. [3]. With the usage of cDNA microarrays, the RNA expression levels of 4028 genes were measured. These stand for about one third of all found fruit fly genes.

In Arbeitman's experiments, 75 sequential sampling points were observed, starting right after fertilization and through embryonic, larval, pupal, and early days of adulthood. The time series data during the embryonic stage are analyzed. The embryonic stage gives us insight into the developmental process, that is, how the fruit fly grows from a zygote to a complex organism with cell specialization. The embryonic data takes the instant of egg lay as the time origin. 30 time points were sampled from  hour to  hours. The greatest common divisor (gcd) for all time intervals is  hour. Therefore  and . The best candidate, Lomb-Scargle, is applied on the data set.

The top 149 genes with the smallest -values are selected and conferred to be periodic with the highest confidence. To remove the effects of DC component, the first two frequency probes are filtered out. The -value is controlled to be less than 0.2. The detailed results are organized into a spreadsheet and provided in the supplementary materials [19]. The majority of genes are associated with a periodicity of about 20 hours, we hypothesize that a portion of them are related to the circadian rhythm. The cell cycle genes are not fully detectable because in the embryonic stage the cells proliferate very fast in minutes, however the implemented sampling rate was not fast enough to capture the phenomenon in the cell cycle.

## 3.3. Discussion of Synchronization Effects

In order to measure a valid sample, the cell culture has to be synchronized, in other words, all cells within the culture should be homogeneous in all aspects, for example, cell size, DNA, RNA, protein, and other cellular contents, and should also mimic the unperturbed cell cycle. Cooper in [29] argued that the ideal synchronization is a mission impossible due to the different dimensions, like cell size and DNA content, that cannot be controlled at the same time. Therefore, current popular synchronization methods, like serum starvation and thymidine block, are only one-dimensional synchronization techniques and fail to achieve a truly global synchronization. Cooper also argued it was fully possible that the discovered periodicity was completely caused by chance or by the specific synchronization method employed. The available fruit fly data set was sampled with the synchronization yielded by the Cryonics method. Cryonics is the low-temperature preservation method of tissues in which all cell activities are believed to be halted. The cells frozen with liquid nitrogen are compared with control cells, that were formaldehyde fixed, to ensure that the cells were at the expected developmental stages during sampling. This synchronization method differentiates itself from the one-dimensional methods employed in [1,2], which have been shown in [29] to present cell cultures that are not actually representative of the cell cycle. Though the damage caused by the freezing was not known, the fly's development assumed true synchronization with the control cells at every developmental check point. This provided enough evidence to consider Arbeitman's data set out of the scope of the issues raised in [29]. Therefore, one can claim with confidence that any discovered periodicity will not have risen from chance fluctuations alone.

## 4. Conclusions

Three of the most representative spectral analysis methods, namely, Lomb-Scargle, Capon, and missing-data amplitude and phase estimation (MAPES) methods, are compared in terms of their performance for detecting the periodically expressed genes in *Saccharomyces cerevisiae*. Lomb-Scargle and Capon methods are computationally efficient while MAPES involves extensive matrix calculations and the iterative expectation maximization (EM) step. Our *in silico* experiments revealed that the simplest method, Lomb-Scargle, outperforms more sophisticated Capon and MAPES. Compared with the other two, Lomb-Scargle method is able to identify more published cyclic genes. This discrepancy between methods is mainly attributed to the data features, such as the small sample size, large proportion of missing samples, and samples highly corrupted by noise. In addition, the computational complexity sacrificed in MAPES for achieving high resolution is not justifiable in the context of gene microarray data. Thus, the computationally simpler methods are more fit for the small sample size scenarios.

The computational results also provide novel insights into the data reported by *Drosophila melanogaster* experiments. A list of 149 genes are identified to express periodically. Their relation with the biological processes are yet to be validated. Our future works also include the development of a comprehensive time-frequency analysis framework for time series microarray data. The small sample size represents another great challenge. Besides, a cross-species study is also desired to examine the relations between fruit fly and homosapiens genes.
